# Cost and cost‐effectiveness analysis of pre‐exposure prophylaxis among men who have sex with men in two hospitals in Thailand

**DOI:** 10.1002/jia2.25129

**Published:** 2018-07-22

**Authors:** Chutima Suraratdecha, Robyn M Stuart, Chomnad Manopaiboon, Dylan Green, Cheewanan Lertpiriyasuwat, David P Wilson, Patcharaporn Pavaputanon, Prin Visavakum, Patama Monkongdee, Thana Khawcharoenporn, Phiphatthananon Tharee, Chonticha Kittinunvorakoon, Michael Martin

**Affiliations:** ^1^ Division of Global HIV and TB Centers for Disease Control and Prevention Atlanta GA USA; ^2^ Burnet Institute Melbourne Victoria Australia; ^3^ Department of Mathematical Sciences University of Copenhagen Copenhagen Denmark; ^4^ Division of Global HV and TB Thailand Ministry of Public Health‐U.S. CDC Collaboration Nonthaburi Thailand; ^5^ Ministry of Public Health Nonthaburi Thailand; ^6^ Division of Infectious Diseases Faculty of Medicine Thammasat University Pathumthani Thailand; ^7^ Lerdsin Hospital Bangkok Thailand

**Keywords:** AIDS, cost, cost‐effectiveness analysis, HIV, modelling, pre‐exposure prophylaxis, Thailand

## Abstract

**Introduction:**

In 2014, the Government of Thailand recommended pre‐exposure prophylaxis (PrEP) as an additional HIV prevention programme within Thailand's National Guidelines on HIV/AIDS Treatment Prevention. However, to date implementation and uptake of PrEP programmes have been limited, and evidence on the costs and the epidemiological and economic impact is not available.

**Methods:**

We estimated the costs associated with PrEP provision among men having sex with men (MSM) participating in a facility‐based, prospective observational cohort study: the Test, Treat and Prevent HIV Programme in Thailand. We created a suite of scenarios to estimate the cost‐effectiveness of PrEP and sensitivity of the results to the model input parameters, including PrEP programme effectiveness, PrEP uptake among high‐risk and low‐risk MSM, baseline and future antiretroviral therapy (ART) coverage, condom use, unit cost of delivering PrEP, and the discount rate.

**Results:**

Drug costs accounted for 82.5% of the total cost of providing PrEP, followed by lab testing (8.2%) and personnel costs (7.8%). The estimated costs of providing the PrEP package in accordance with the national recommendation ranges from US$223 to US$311 per person per year. Based on our modelling results, we estimate that PrEP would be cost‐effective when provided to either high‐risk or all MSM. However, we found that the programme would be approximately 32% more cost‐effective if offered to high‐risk MSM than it would be if offered to all MSM, with an incremental cost‐effectiveness ratio of US$4,836 per disability‐adjusted life years (DALY) averted and US$7,089 per DALY averted respectively. Cost‐effectiveness acceptability curves demonstrate that 80% of scenarios would be cost‐effective when PrEP is provided solely to higher‐risk MSM.

**Conclusion:**

We provide the first estimates on cost and cost‐effectiveness of PrEP in the Asia‐Pacific region, and offer insights on how to deliver PrEP in combination with ART. While the high drug cost poses a budgeting challenge, incorporating PrEP delivery into an existing ART programme could be a cost‐effective strategy to prevent HIV infections among MSM in Thailand.

## 1 Introduction

HIV pre‐exposure prophylaxis (PrEP) can reduce sexual and parenteral transmission of HIV. The World Health Organization recommends PrEP as part of a comprehensive HIV prevention package including HIV testing, counselling, condoms, lubricants, antiretroviral therapy (ART) for partners of people living with HIV, voluntary medical male circumcision and harm reduction interventions for people who use drugs 1. Oral PrEP has been shown to be cost‐effective in settings where the HIV incidence is >3 per 100 person‐years and in some settings at lower incidence 1, 2, 3, 4, 5. Although the literature suggests that drug costs of oral PrEP are lower than antiretroviral (ARV) drug costs on the basis of cost per dose and duration of use, funding the high cost of PrEP on top of other prevention programmes remains one of the main challenges, especially in resource‐constrained settings 6, 7, 8, 9.

Thailand included PrEP as an HIV prevention tool within their 2014 national HIV Treatment and Prevention Guidelines 10. This is to complement key components of their National Operational Plan for Ending AIDS 2015 to 2019 including increasing HIV testing coverage, facilitation of those who test positive to initiate and stay on treatment, and to provide HIV prevention services to those who test negative 11. In Thailand, PrEP services are either available free of charge or at low‐cost at several locations 12. As of June 2017, an estimated 1044 men having sex with men (MSM) had received services from the fee‐based PrEP‐30 demonstration project (1000 Thai Baht (THB) per month, around US$30), implemented by the Thai Red Cross Society 12. Around 1000 MSM and transgender (TG) women have enrolled in the Princess PrEP project, which started in 2016 under the Thai Royal patronage 12. Another 100 MSM and TG women were enrolled in PrEP2START, a government‐initiated PrEP scale‐up programme, which has been implemented in eight HIV high‐burden provinces since November 2016, and which aims to strengthen the public health system and to enhance capacity of healthcare professionals in providing PrEP to MSM and TG 13. Under the PrEP2START programme drugs are provided free of charge, but participants contributed to laboratory services ranging from US$11 to US$28 annually 13.

Evidence suggests that PrEP programmes have experienced both challenges and successes. An assessment of the PrEP‐30 project in early 2016 reported no new infections among PrEP users during the first year of project implementation 13. The Princess PrEP project showed a 52% retention rate at six months with a 97% adherence rate of ≥4 tablets per week (N = 671) 12. Another study reported willingness to use PrEP among MSM and TG women ranging from 36% to 41%, with 65% willing to pay a maximum of US$21.40 per month for PrEP 13. However, wider implementation has been limited and evidence on the epidemiological and economic impact, and costs of PrEP implementation is not available. While demonstration projects indicate feasibility of PrEP implementation, significant challenges for scale‐up of PrEP services in Thailand will include securing the financial resources to make PrEP more widely available and mobilization of human resources to prescribe PrEP in a timely manner 14.

The objectives of this study were to assess the cost of providing oral PrEP to MSM, and to estimate the epidemiological impact and cost‐effectiveness of oral PrEP for this target group. This study adds new evidence to the existing literature by estimating primary costs for delivering PrEP in facility‐based settings and investigating the impact of potential parameters, including programme effectiveness, uptake, and condom use on the cost‐effectiveness of PrEP provision.

## 2 Methods

This study was approved by the Thailand Ministry of Public Health (MOPH) Ethical Review Committee and as a non‐research programme evaluation by the U.S. Centers for Disease Control and Prevention (CDC). All procedures were in compliance with the Helsinki Declaration, the Code of Federal Regulations, Title 45, Part 46 (45 CFR §46), and local ethical and legal requirements. A written enrolment consent form was sought from potential participants in the cohort study. Staff consent was not required.

### 2.1 Prospective observational cohort study

In May 2015, the Thailand MOPH in collaboration with the CDC launched the Test, Treat, and Prevent HIV Programme to identify barriers to the immediate initiation of ART and the use of PrEP. Participants were enrolled and followed from 1 May 2015 to 30 April 2018 including 1880 Thai MSM and TG from the Khon Kaen, Lerdsin, Srinagarind University, Thammasat University, and Udon Thani Hospitals across four provinces (Bangkok, Khon Kaen, Pathum Thani and Udon Thani). PrEP was also offered at Lerdsin and Thammasat Hospitals 15.

### 2.2 Cost analysis

We conducted a retrospective cost analysis of the Test, Treat and Prevent HIV Programme from 1 June 2015 to 31 May 2016 to derive the cost associated with HIV testing and PrEP services from a provider perspective. Costs of HIV testing and PrEP services were collected by programmatic activity (HIV testing and counselling, PrEP initiation and PrEP visit (adherence counselling, physical check, clinic visit, prescribing drugs and blood draw)), by input type (personnel, drugs and commodities, supplies, test kits, laboratory testing including creatinine, hepatitis B, syphilis screening, biochemistry and haematology), and by source of support (cohort study or MOPH). We used a time and motion analysis to measure the average time health and lab staff spent on each programmatic activity session. To calculate personnel costs, we collected information from facility and project staff on their salaries and allowances, and assumed staff worked eight hours per day for 20 days each month. We based the cost of supplies and equipment associated with laboratory testing on price per test using the quotes from the suppliers of lab reagents multiplied by the number of tests conducted during the study. The price paid per test covers the cost of the laboratory equipment, services, reagents and consumables for the term of the agreement excluding capital expenditures. The cost of drugs and commodities was determined by multiplying the MOPH unit price with the quantities used. The study excluded costs associated with HIV‐related morbidity and mortality, as well as morbidities and mortalities stemming from adverse events associated with care and treatment, costs borne by the health system, higher‐level overhead costs, and building maintenance and utility costs. Data on beneficiary volume were extracted from the outcomes of the cohort study to derive the unit cost per‐person‐per‐year (ppy) by dividing the total cost of the programme by the number of PrEP participants. Data collection was conducted from October to December 2016. All costs were collected in THB and converted to U.S. dollars (USD) at the market exchange rate (1 USD = 35.42174 THB) for the period the cost was incurred.

### 2.3 Impact and cost‐effectiveness analysis

To estimate the epidemiological impact of providing PrEP to MSM, we used the Optima HIV tool (v2.6.6, available at http://hiv.optimamodel.com), which is a compartmental model of HIV transmission and disease progression linked to a programmatic response module for estimating the epidemiological and economic impact of interventions 16, 17. Of the approximately 571,000 MSM in Thailand 18, about one‐third are characterized as being high‐risk on the basis of having engaged in condomless sex with casual or known HIV‐positive partners 19, 20. We created a model of the MSM population in Thailand disaggregated into low‐risk and high‐risk groups and populated the model with available country‐specific data on population sizes, sexual behaviour, and testing rates, disaggregated by risk group where possible (Table 1). Non‐context specific parameters were also used to inform the model as documented in the Optima HIV user guide 21. We calibrated the model to historical HIV prevalence estimates to produce baseline estimates of the expected number of new HIV infections over the period from the beginning of 2017 to the end of 2022 in the absence of PrEP.

**Table 1:** Key parameters for the epidemiological model

Parameter	Value	Source and notes
Condom use among MSM with casual partners	82%	2015 18
Condom use among MSM with regular partners	66%	Condom use with all male steady partners, value consistent from 2005 and 2007 22
Per‐act transmission probabilities	Varying	21
Efficacy of interventions	Varying	21
Percentage MSM tested for HIV in the last 12 months	30.85%	2015 23
HIV prevalence among MSM		
High‐risk	11.6%	2017 23
Low‐risk	5.2%	2017 23
Population size		
High‐risk	155,149	2017 23
Low‐risk	362,055	2017 23

MSM, men having sex with men.

We assumed a five‐year implementation period (fiscal period 2017‐2018 to 2021‐2022) and measured HIV infections and disability‐adjusted life years (DALYs) averted under a range of different scenarios around PrEP provision. DALYs were calculated from the onset of infection and cost and effects were discounted at an annual rate ranging from 1.5% to 4.5%. Disability weights were obtained from the literature 21.

A knowledge of the baseline level of ART coverage among MSM is essential in assessing the impact and cost‐effectiveness of providing PrEP. In 2014, ART coverage among MSM was reported as 7% 18, but this is likely to be an underestimate as it only includes people who self‐reported as MSM. The ART coverage among the total population of Thailand was approximately 60% 24. To account for the uncertainty surrounding coverage of ART among MSM, we assumed baseline ART coverage of 30% and allowed coverage to vary between the estimates for MSM and for the total population. We further assumed that the scale‐up in ART coverage over the five‐year PrEP implementation will not exceed 20 percentage points. For example, if the baseline ART coverage is assumed to be 7%, we allowed ART coverage levels in 2021 to 2022 to range between 7% and 27%. With the baseline ART coverage of 60%, we allowed 2021 to 2022 coverage levels to range between 60% and 80%, with the upper range corresponding to the achievement of the UNAIDS 90‐90‐90 treatment targets.

We considered two core sets of scenarios based on the parameters listed in Table 2, one in which PrEP will be provided to all MSM and one in which PrEP will only be provided to high‐risk MSM. The uptake of PrEP in each scenario is linked to ART coverage levels, with PrEP uptake in each scenario ranging from 0% up to the assumed levels of ART coverage in 2021 to 2022. We consider the possible impact of PrEP on sexual disinhibition by modelling the impact of reductions in condom use among MSM. While we do not specifically model the effects of other changes in sexual behaviour (for example, an increase in the number of partners), we expect that the results would be similarly sensitive to other forms of sexual disinhibition 25.

**Table 2:** Model parameters for PrEP implementation scenarios

Parameter	Value	Range	Notes
PrEP programme effectiveness	75%	50% to 95%	The variation in programme effectiveness reflects uncertainty in both drug efficacy and adherence/retention.
Baseline ART coverage among MSM in 2017/18	30%	7% to 60%	ART coverage among MSM in 2014 was reported at 7% 18 (likely to be underestimated as this only includes ART clients who self‐reported as MSM). ART coverage was approximately 60% among the total population 24. We used a default value of 30% and allowed coverage to vary between the estimates for MSM and for the total population.
ART scale‐up relative to baseline	10 ppts increase	0 to 20 ppts increase	We set ART coverage among MSM in 2021/22 relative to the value in 2017/18. Our default assumption is for a slight scale‐up relative to baseline; we contrast this with maintenance of baseline levels and a larger increase. Since our baseline ART coverage assumptions range up to 60%, an assumption of a 20 percentage point scale‐up encompasses the accomplishment of the UNAIDS 90‐90‐90 targets.
PrEP uptake among high‐risk MSM	10 ppts lower than ART coverage levels	From 0% up to ART coverage levels	As a baseline, we assume that PrEP uptake will be 10 percentage points lower than ART coverage levels. We consider uptake values ranging from 0% up to ART coverage levels.
PrEP uptake among low‐risk MSM			
Scenario: PrEP is provided to all HIV‐negative MSM	10 ppts lower than ART coverage levels	From 0% up to ART coverage levels	
Scenario: PrEP is provided to HIV‐negative high‐risk MSM	0%	No range	No uptake among low‐risk MSM
Percentage reduction in condom use for those receiving PrEP	10%	0% to 20%	Moderate reduction in condom use in the moderate scenario, no reduction in the ambitious scenario and 20% reduction in the conservative scenario.
PrEP unit cost ppy	US$222.18	US$180 to US$310	
Annual discounting rate	3%	1.5% to 4.5%	

MSM, men having sex with men; PrEP, pre‐exposure prophylaxis; ART, antiretroviral therapy; ppy, per‐person‐per‐year; ppts, percentage points.

We ran 60,000 model simulations, in which samples of each of the eight parameters listed in Table 2 were drawn from uniform distributions over their allowable ranges, and calculated the total number of new HIV infections and DALYs that were estimated over the implementation period for each simulation.

For the cost‐effectiveness analysis, we calculated the cost associated with each scenario by disaggregating the cost of PrEP and ART. To derive the cost of the PrEP intervention, we multiplied the number of HIV‐negative MSM by the percentage of MSM receiving PrEP and then by the unit cost ppy of the PrEP programme. We multiplied the number of HIV‐positive MSM by the percentage of MSM receiving ART, and then by the unit cost ppy of the ART programme to estimate the cost of ART provision. The median cost per person on ART per year including ARV medicines of US$369.57 was obtained from the Test, Treat and Prevent HIV Programme costing study from the same cohort 26. We used a cost‐effectiveness threshold of US$17,449, equal to three times the gross domestic product (GDP) per capita 27 to determine the cost‐effectiveness of PrEP 28.

## 3 Results

### 3.1 Annual PrEP costs

From 1 June 2015 to 31 May 2016, 366 HIV‐negative MSM and TG participants were recruited at Lerdsin and Thammasat University Hospitals to participate in the PrEP sub‐study. Of the 366 participants, 163 (44.5%) accepted PrEP free‐of‐charge for 12 months, and this was used to determine the acceptance rate and to assess costs. Drug costs accounted for 82.5% of the total annual costs associated with PrEP provision (Table 3). HIV testing costs were US$824 per year. The unit cost ppy, including HIV testing for all clinic visits and the cost of project staff who provided PrEP was US$129. Project staff were hired to support PrEP services, but if the PrEP programme is implemented, project staff will be replaced by MOPH staff. The average unit cost of providing PrEP ppy without project staff is US$128. We assumed that the unit cost of providing PrEP to MSM will be the same as that derived for MSM/TG from the cohort study.

**Table 3:** Distribution of total annual costs (USD) associated with PrEP initiation and clinic visits by input type

Input type	Cohort study (including MOPH and project staff)	Projection (MOPH staff only)
Personnel (health and laboratory staff)	$1,452 (8.4%)	$1,337 (7.8%)
Lab supplies and reagents	$1,406 (8.2%)	$1,406 (8.2%)
PrEP drugs	$14,106 (82.0%)	$14,106 (82.5%)
Other supplies	$242 (1.4%)	$242 (1.4%)
Total	$17,206	$17,091

USD, U.S. dollars; PrEP, pre‐exposure prophylaxis; MOPH, Ministry of Public Health.

We projected the cost if each participant took PrEP continuously over the 12‐month study period, attended clinic and monitoring visits, and received HIV and lab tests following recommended PrEP programme guidelines 29. We applied a micro costing approach to estimate the unit cost ppy and quantities for personnel, HIV and lab testing, and drugs for each package (Table 4). We applied a 0.7% proportion of overhead costs from a similar existing study in Thailand 30 to the unit cost. In the cohort study, demand creation activities were included in each counselling session. Personal communications with a programme officer from a pilot PrEP project conducted previously in Thailand indicated that the spending for demand creation activities for both HIV testing and PrEP accounted for approximately 22% of the project budget. Although the amount included demand creation activities for PrEP (inclusive of activities during the counselling session) and for HIV testing, we applied this proportion to the unit cost to reflect additional demand creation activities that may be offered. The cost to implement the PrEP programme ranges from US$222.89 to US$310.99 ppy depending on the package options and demand creation activities chosen.

**Table 4:** Unit cost (USD) of PrEP recommended package by option per person per year

Cost category	Option 1^a^	Option 2^b^
Personnel (health and laboratory staff) to provide HIV testing and counselling, one visit for initial PrEP counselling and recruitment, six visits of maintenance support (counselling), four additional HIV tests, two creatinine tests, one Hepatitis B surface antigen (HBs Ag) test and two upgraded STIs screenings (option 2 only)	$24.66	$25.63
Supplies (HIV testing and lab testing) for five HIV tests (initiating testing and four additional tests), two creatinine tests, one HBs Ag test and two upgraded STIs screenings (option 2 only)	$10.36	$41.41
Tenofovir/Emtricitabine (TDF/FTC) (12 bottles: 1 pill per day)	$186.33	$186.33
Total unit cost ppy	$221.34	$253.37
Total unit cost ppy with overhead 0.7%	$222.89	$255.14
Total unit cost ppy with overhead 0.7% and 22% demand creation activities	$271.59	$310.88

USD, U.S. dollars; PrEP, pre‐exposure prophylaxis; ppy, per‐person‐per‐year.

^a^Option 1 package includes one visit for initial PrEP counselling and recruitment, four additional HIV tests, two tests for creatinine, one HBs Ag test, 12 months TDF/FTC combination (tenofovir disoproxil fumarate/emtricitabine), six visits for maintenance support (counselling); ^b^Option 2 package includes the option 1 package plus two times upgraded STIs screening (chlamydia, gonorrhoea, syphilis rapid test, nucleic acid amplification test).

### 3.2 Impact and cost‐effectiveness of PrEP

In Table 5 we present results on the number of HIV infections averted, lifetime treatment costs averted, and the incremental cost‐effectiveness ratio (ICER) for both HIV infections and DALYs averted over the five‐year implementation period. Results are presented for two core implementation strategies: PrEP for high‐risk MSM only and PrEP for all MSM, and are based on the scenarios generated using the parameter values shown in Table 2. Comparing these two strategies, we estimate that providing PrEP to all MSM would have a greater epidemiological and economic impact than providing PrEP to high‐risk MSM only, with almost 2.5 as many HIV infections averted (1368 *vs*. 555) and more than twice the discounted lifetime treatment costs averted (US$9.84 million *vs*. US$3.99 million). However, providing PrEP only to high‐risk MSM would be approximately 32% more cost‐effective than providing it to all MSM, with ICERs of US$4,836 and US$7,089 per DALY averted respectively. Applying the cost‐effectiveness threshold of three times GDP per capita of US$17,449, the ICERs per DALY averted for both targeting strategies demonstrate the cost‐effectiveness of PrEP services.

**Table 5:** Incremental cost‐effectiveness ratios (ICERs) for core scenarios, based on the parameter values given in Table 2

Scenario name	Cost	Effectiveness			ICERs	
	Total five‐year programme cost (millions)	DALYs averted	HIV infections averted	Lifetime treatment costs averted (million)	$/DALY averted	$/HIV infection averted
PrEP provided to high‐risk MSM	$41.99	7,857	555	$3.99	$4,836.00	$68,468.00
PrEP provided to all MSM	$147.14	19,368	1368	$9.84	$7,089.00	$100,367.00

DALYs, disability‐adjusted life years; $, U.S. dollars.

### 3.3 Sensitivity analysis

**Figure 1:**
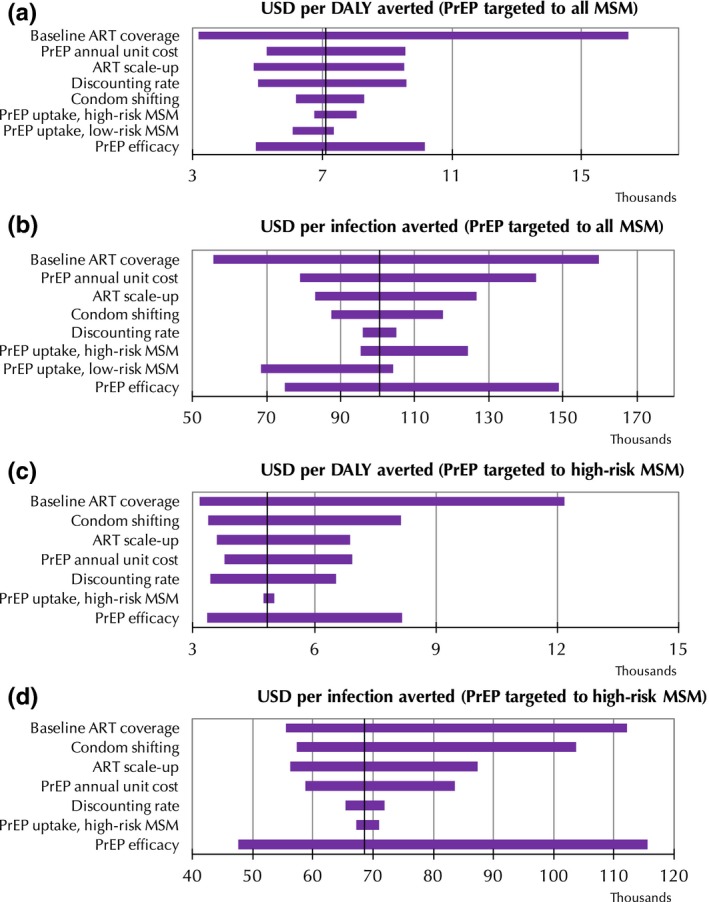
Tornado diagrams of univariate sensitivity analysis.

Figure 1 presents tornado diagrams summarizing the one‐way sensitivity analysis of the ICERs for both HIV infections and DALYs averted based on the model parameters defined in Table 2. The ICERs are particularly sensitive to both baseline ART coverage and the ART scale‐up rate. Higher ART baseline coverage and a more rapid scale‐up of ART would make the PrEP programme relatively less cost‐effective. These results are also sensitive to the PrEP programme unit cost. In scenarios where PrEP was only provided to high‐risk MSM with all other parameter values as indicated in Table 2, the ICER for HIV infections averted is estimated to lie between US$58,670 and US$83,587, while the ICER for DALYs averted is estimated to lie between US$3,767 and US$6,946. We estimate that these ranges would increase to US$78,855 to US$142,828 and US$5,266 to US$9,559 respectively if PrEP was provided to all MSM. We also find that the scenario for providing PrEP only to high‐risk MSM, if condom use while using PrEP were maintained, would be particularly cost‐effective.

**Figure 2:**
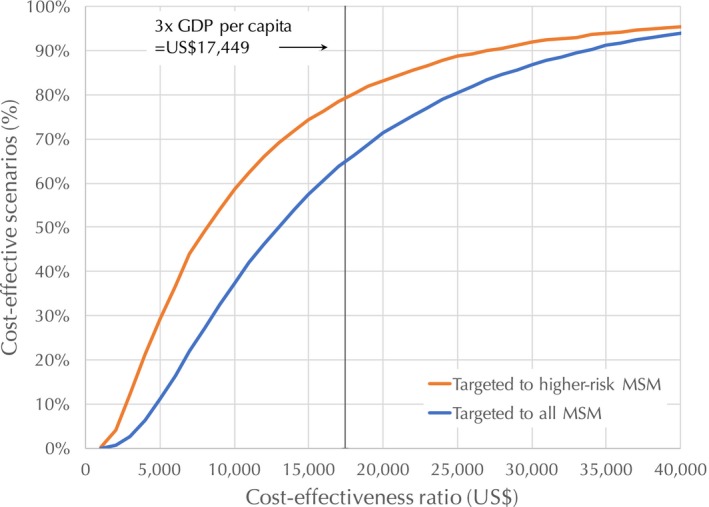
Cost‐effectiveness acceptability curve for pre‐exposure prophylaxis strategies.

Using a cost‐effectiveness threshold of three times GDP per capita, an estimated 80% of PrEP implementation scenarios (as constructed using the parameter values from Table 2) are calculated to be cost‐effective if the PrEP programme is provided solely to high‐risk MSM, compared to 66% for all MSM (Figure 2). The minimum and maximum ICERs for DALYs averted are estimated to be US$702 (assuming a PrEP unit cost of US$180, 95% PrEP programme effectiveness, no reduction in condom usage, ART coverage remaining constant at 7%, and a 1.5% discount rate) and US$218,802 ($310 PrEP unit cost, 55% PrEP programme effectiveness, 20% reduction in condom usage, 60% ART coverage increasing to 80% by 2021 to 2022, and a 4.5% discount rate) respectively.

## 4 Discussion

There is strong evidence for the safety and efficacy of PrEP among MSM and other high‐risk groups 2, 31. However, the evidence on cost‐effectiveness of PrEP is mixed, with results varying depending on the unit cost of PrEP, population targeted, and the broader health system context 32. This suggests that country‐specific cost‐effectiveness analyses may be needed to inform national policy on PrEP implementation strategies. Accordingly, a growing number of studies have been published in recent years to fill this gap. We searched PubMed from 1 January 2013 to 11 October 2017 with the terms “HIV” AND (“PrEP” OR “PrEP”) AND (“cost” OR “cost‐effectiveness”) with the goal of identifying any new studies that have appeared since the 2013 meta‐analysis of PrEP cost‐effectiveness modelling studies 32. The search retrieved 149 abstracts, of which 21 provided country‐specific estimates of the cost‐effectiveness of various PrEP implementation strategies 6, 8, 9, 33, 34, 35, 36, 37, 38, 39, 40, 41, 42, 43, 44, 45, 46, 47, 48, 49. This study is not only the first to provide data and evidence on the costs and cost‐effectiveness of providing oral PrEP to MSM in Thailand, but is also the first such study from the Asia‐Pacific region.

The unit cost of the PrEP programme in the cohort study was lower than that of the recommended package because not all study participants attended clinic visits quarterly and took PrEP continuously, and the visit and testing schedules were different between the cohort study and recommendation. Since PrEP was offered as an integrated service to ART services in the study, the cost difference attributed to project staff was less than US$1 per person‐year. Consistent with findings from other countries 32, drug costs represent the majority of PrEP programme costs. If the PrEP programme could be delivered according to the recommended package at a unit cost of US$222.89 ppy and provided to high‐risk MSM, we estimate that it would be a very cost‐effective package, with an estimated ICER of US$4,836 per DALY averted, lower than Thailand's per‐capita GDP of US$5,816. Through a sensitivity analysis we estimate that if the PrEP programme is only provided to high‐risk MSM, 80% of the scenarios that we considered would be cost‐effective, compared to 66% if the programme is provided to all MSM.

There are several limitations to this study. The cost associated with community‐based outreach activities, such as demand creation and peer support groups (e.g. to improve high‐risk MSM enrolment or adherence) were not available and were not implemented in the cohort study. We applied the cost of demand creation activities based on the information from another project and included overhead costs obtained from another study as a proxy to define the range of unit costs. The project engaged senior facility staff during implementation, and the cost per person per year may be lower if less senior staff were deployed. Laboratory testing requirements varied among the facilities included in this study. The proportion of lab costs from the recommended PrEP package were much less than the project costs due to the type and quantity of tests used in this study. In our study, most tests were conducted quarterly and other tests, such as biochemical tests were also included. Our analysis did not consider new diagnostics, for example HIV self‐testing, which may lead to further reductions in cost. Costs could be reduced if PrEP were offered on demand or for less than 12 months. Due to limited resources, we did not collect data on cost to clients, which would be useful to determine the potential financial barriers and copayment. Our sensitivity analysis attempted to account for cost variation due to outreach activities supporting adherence, drug prices, and duration of PrEP uptake. Not all possible factors that may impact the cost‐effectiveness of PrEP were included – for example, we did not account for potential cost savings or DALYs averted associated with screening for sexually transmitted infections, nor for the possible changes in the number of partners that might result from PrEP uptake. Instead we opted to model the impact of sexual disinhibition as impacting condom use only. We expect that any additional changes in sexual behaviour would impact the cost‐effectiveness of PrEP in a comparable way to the impact of changes in condom use. While we did not consider the budget‐impact analysis, which will further guide the resource allocation decision, we have attempted to cost the PrEP programme and have extrapolated the costs of the recommended package.

## 5 Conclusions

This analysis provides insights on various PrEP delivery strategies, as well as an analysis of some of the factors that influence PrEP impact and cost‐effectiveness. These findings show that the cost‐effectiveness ratios are sensitive to ART coverage and PrEP programme effectiveness. A PrEP strategy targeting high‐risk MSM if condom use does not decrease was found to be the most cost‐effective intervention. Evidence from both Thailand 13 and from other settings has consistently shown no difference in condom use with uptake of PrEP 31, so there is reason to be hopeful that the best‐case scenarios provided in this study would be reflected if PrEP were implemented. While elevated PrEP drug costs will continue to pose a budgeting challenge, our results indicate that providing PrEP to high‐risk MSM is likely to be a cost‐effective HIV prevention intervention.

## Competing interests

The authors declare no competing interests.

## Authors’ contributions

CM, CL, PP, MM developed the cohort study. CS, DG, CM and MM developed the costing study design. CS, RS and DW developed the cost‐effectiveness analysis. CS and RS produced the results and were the lead writers of the manuscript. PV, PM, PT, TK and CK collected the data. All authors reviewed and contributed to the final manuscript and have read and approved the final version.
